# Cytochrome P450 2J2 inhibits the proliferation and angiogenesis of retinal vascular endothelial cells by regulating the Notch signaling pathway in a hypoxia-induced retinopathy model

**DOI:** 10.1080/21655979.2021.1994722

**Published:** 2021-11-30

**Authors:** Jing Zhang, Qi Xiong, Lin Yang, Yanni Xue, Min Ke, Zhi Li

**Affiliations:** Department of Ophthalmology, Zhongnan Hospital of Wuhan University, Wuhan, China

**Keywords:** CYP2J2, retinal vascular endothelial cells, Notch pathway, hypoxia-induced retinopathy

## Abstract

Retinopathy of Prematurity (ROP), a type of retinal neovascularization in premature infants, has become a serious problem that drastically affects the quality of life of premature infants. ROP is associated with angiogenesis and neovascularization. Here, we aimed to explain the function and latent roles of Cytochrome P450 2J2 (CYP2J2) in hypoxia-induced retinopathy in retinal vascular endothelial cells (HRVECs). HRVECs were stimulated with hypoxia for 24 h to establish an *in vitro* retinopathy model. Cell viability and migration were evaluated using 3-(4,5-dimethyl-2-thiazolyl)-2,5-diphenyl-2-H-tetrazolium bromide (MTT) and Transwell assays, respectively. Protein and gene expression was determined by reverse transcription quantitative real-time polymerase chain reaction (RT-qPCR) and Western blot analysis. We observed that pcDNA3.1(+)-CYP2J2 promoted CYP2J2 and Jagged1 expression, while Dll4 was down-regulated in hypoxia-stimulated HRVECs. Additionally, pcDNA3.1(+)-CYP2J2 inhibited HRVEC viability, reduced PCNA expression, and inhibited the migration of HRVECs. Further, the Notch pathway was inhibited in the Hypoxia+pcDNA3.1(+)-CYP2J2 group. Opposite results were observed upon Terfenadone treatment in hypoxia induced HRVECs. Finally, our findings further verified that DAPT promotes the effects of CYP2J2 on cell viability, migration, and Notch signaling in hypoxia-induced HRVECs, while EDTA reversed the inhibitory effects of CYP2J2 on hypoxia-induced HRVECs. In conclusions, CYP2J2 was found to inhibit the viability and angiogenesis of HRVECs by inhibiting Notch signaling in a hypoxia-induced retinopathy model.

## 1. Introduction

The classification of retinopathy is complex and includes retinal detachment, diabetic retinopathy, endophthalmitis, and retinopathy of prematurity (ROP) [1–3]. ROP is a type of retinal neovascularization in premature infants. In severe cases, ROP can cause retinal proliferation, detachment, and even blindness [4]. The incidence of ROP from 23–26 weeks can be as high as 30–40%, making ROP a key problem that seriously affects the quality of life of premature infants [5]. In the past decade, with the widespread use of VEGF drugs, intravitreal injection of anti-VEGF has become an effective method for early treatment of ROP [6]. However, blocking VEGF alone does not completely prevent retinal neovascularization and instead may inhibit normal vascular development and aggravate neurodegeneration [7]. Simultaneously, it can also produce adverse reactions such as endophthalmitis and systemic anti VEGF risk [7]. Therefore, the key to improving therapeutic treatment of ROP is to explore safer and more effective methods to inhibit neovascularization.

Arachidonic acid (AA), one of the most abundant and active polyunsaturated fatty acids, was found to be metabolized by cytochrome P450 (CYP450) oxidase to alleviate hypoxic-ischemic injury and regulate angiogenesis [8]. CYP2J2 is a type of surface oxidase in CYP450 oxidase that plays an important role in regulating blood vessels. Studies have shown that CYP2J2 gene polymorphisms can affect the occurrence and development of ischemic stroke by modulating transcriptional activity [9]. In addition, CYP2J2 produced epoxyeicosatrienoic acids attenuate ischemia-reperfusion (I/R)-induced acute kidney injury by activating the SIRT1-FoxO3a pathway [10]. However, whether CYP2J2 can also regulate blood vessels in the retinal vascular system requires further investigation.

Notch signaling affects many processes of normal morphogenesis, including pluripotent progenitor cell differentiation, apoptosis, cell proliferation, and cell boundary formation [11]. This pathway is highly conserved and necessary for normal embryonic development, tissue homeostasis, and angiogenesis. In the central nervous system, Notch signaling plays an important role in regulating angiogenesis. Wang et al. found that TRIM28 regulates sprouting angiogenesis through the VEGFR-DLL4-Notch signaling circuit [12]. Notch signaling also promotes angiogenesis and improves cardiac function after myocardial infarction [13]. In an oxygen-induced retinal model, Dll4 expression is significantly increased in the neovascularization cluster, and inhibition of Dll4/Notch may lead to faster revascularization of the ischemic part of the retina [14], although the mechanism remains unclear.

We hypothesized that CYP2J2 might affect hypoxia-induced HRVECs via regulating Notch signaling pathway. Therefore, this study was designed to reveal the underlying roles of CYP2J2 in retinal vascular endothelial cells (HRVECs) after hypoxia treatment and explore its underlying mechanisms.

## 2. Materials and methods

### 2.1 Establishment of a hypoxia-induced model of ROP

HRVECs were purchased from American Tissue Culture Colection (ATCC) and maintained in DuIbecco’s modified eagIe’s medium (DMEM) medium (Gibco, CA, USA) supplemented with 10% fetal bovine serum (FBS, Gibco), 100 mg/ml streptomycin, and 100 IU/ml penicillin in a humidified incubator with 5% CO_2_ at 37°C. Then, HRVECs were cultured in hypoxia (1% O_2_, 5% CO_2_, 94% N_2_) for 24 h to establish an *in vitro* hypoxia-induced retinopathy model. Meanwhile, HRVECs were cultured under normal conditions (20% O_2_, 5% CO_2_, 75% N_2_) for 24 h as a Normal control [15].

### 2.2 Cell transfection

pcDNA3.1(+)-GFP and pcDNA3.1(+)-CYP2J2 were obtained from GenePharma (Shanghai, China). pcDNA3.1(+)-GFP and pcDNA3.1(+)-CYP2J2 were transfected into HRVECs using Lipofectamine 2000 (Invitrogen; Thermo Fisher Scientific, Inc.), according to the manufacturer’s instructions.

### 2.3 Cell treatment

To explore the effects of CYP2J2 down-regulation on hypoxia-induced HRVECs, hypoxia-induced HRVECs were treated with a CYP2J2 inhibitor (Terfenadone; 0.2 µM) for 24 h.

To explore the effects of Notch1 pathway hypoxia-induced HRVECs, hypoxia-induced HRVECs were treated with Notch1 inhibitor, N-[N-(3,5-difuor-ophenacetyll-alanyl)]-S-phenylglycine t-butylester (DAPT; 10 µM), or Notch1 agonist (EDTA; 5 mM) for 24 h.

### 2.4 RT-qPCR analysis

Following treatment, the levels of CYP2J2, Dll4, Jagged1, Notch1, Hes1/5, Hey1, and GAPDH were measured by RT-qPCR . RNA was isolated from HRVECs using the RNA-isolation kit (Life Technologies, USA) following the manufacturer’s protocol. Total RNA was reverse transcribed to cDNA using the PrimeScript RT Reagent Kit (TaKaRa, China) following the manufacturer’s instructions, and RT-qPCR was performed using the SYBR PrimeScript RT-PCR Kit (TaKaRa) with an ABI 7500 Real-Time PCR System (Agilent Technologies, USA). Target gene expression was quantified using the 2^−ΔΔCt^ method [16]. Primer sequences were listed as following:

CYP2J2, forward 5ʹ-TCCATCCTCGGACTCTCCTAC-3ʹ;

Reverse5ʹ-GTCACCAAGCTCCAAGCTAAAA-3ʹ;

Dll4,forward5ʹ-GTCTCCACGCCGGTATTGG-3ʹ;

Reverse5ʹ-CAGGTGAAATTGAAGG GCAGT-3ʹ;

Jagged1, forward 5ʹ-ACTGCTCACACCTGAAAGACCAC-3ʹ;

Reverse5ʹ-AGGACCACAGACGTTGGAGGAAA-3ʹ;

Notch1, forward 5ʹ-AGGACCTCATCAACTC ACACGC-3ʹ;

Reverse 5ʹ-TCTTTGTTAGCCCCGTTCTTCA G-3ʹ;

Hes1, forward 5ʹ-AGGCGGACATTCTGGAAA TG-3ʹ;

Reverse 5ʹ-CGGTACTTCCCCAGCACAC TT-3ʹ;

Hes5, forward *5ʹ-CTGGAGATGGCCGTCAG CTACCTG-3*ʹ;

Reverse *5ʹ-GAGTAGCCCTCGCTGTAGTCCT G-3*ʹ;

Hey1, forward 5ʹ-CTGCAGATGACCGTGGA TCA-3ʹ;

Reverse 5ʹ-CCAAACTCCGATAGTCCATAG CA-3ʹ;

GAPDH, forward 5ʹ-TCATGGGTGTGAAC CATGAGAA-3ʹ;

Reverse 5ʹ-GGCATGGACTGTGGTCATGA G-3ʹ.

### 2.5 Western blot assay [17]

After treatment, proteins were harvested from HRVECs with radio-immunoprecipitation assay (RIPA) lysis buffer (Beyotime). Protein concentration was determined using a BCA Protein Assay kit (Invitrogen, USA). Proteins (40 μg proteins per lane) were then resolved by 10% sodium dodecyl sulfate polyacrylamide gel electrophoresis (SDS-PAGE) and transferred onto polyvinylidene fluoride (PVDF) membranes. After blocking with 5% skim milk in Phosphate Buffer Solution-0.1% Twen-20 (PBST) for 1 h, the membranes were incubated in primary antibodies against GAPDH (37 kDa; cat. no. 5174; 1: 1000 dilution; Cell signaling Technology), VEGFA (23 kDa; cat. no. ab46154; 1: 1000 dilution; Abcam), PCNA (36 kDa; cat. no. 13110; 1: 1000 dilution; Cell signaling Technology), CYP2J2 (57 kDa; cat. no. Sc-137127; 1: 1000 dilution; Santa Cruz Biotechnology), Dll4 (80 kDa; cat. no. 96406; 1: 1000 dilution; Cell signaling Technology), Jagged1 (180 kDa; cat. no. 70109; 1: 1000 dilution; Cell signaling Technology), Notch1 (120 kDa; cat. no. 3608; 1: 1000 dilution; Cell signaling Technology), Hes1 (30 kDa; cat. no. 11988; 1: 1000 dilution; Cell signaling Technology), Hes5 (18 kDa; cat. no. ab194111; 1: 1000 dilution; Abcam), or Hey1 (33 kDa; cat. no. ab154077; 1: 1000 dilution; Abcam) overnight at 4°C. Then, the membranes were incubated with Anti-rabbit IgG, HRP-linked Antibody (cat. no. 7074; 1: 2000 dilution; Cell signaling Technology) for 1 h. Finally, the protein bands were visualized using electrochemiluminescence (ECL) detection system reagents (Pierce, USA) and quantified using Image J Software.

### 2.6 MTT assay [18]

After treatment, HRVECs were cultured in 96-well plates at 37°C. Then, cells were treated with 10 μl MTT (5 mg/ml) solution and continuously incubated for 4 h. After incubation, the culture medium was removed and 150 µl of DMSO was added to dissolve the formazan product in the dark for 10 min. Finally, the optical density (OD) at 570 nm was measured using a microplate reader (BioTek, USA) after vibration mixing following the manufacturer’s protocol. Cell viability (%) = (OD value of the experiment group – OD value of the blank group)/(OD value of the control group – OD value of the blank group) × 100%.

### 2.7 Transwell migration assay [19]

After treatment, HRVECs were cultured in DMEM medium and seeded into the upper chamber of a transwell chamber, with the lower chamber containing DMEM medium containing 10% FBS. After incubating at 37°C with 5% CO_2_ for 48 h, cells remaining on the upper chamber (not migrated) were removed and cells on the lower chamber were fixed with 4% paraformaldehyde and stained with 0.1% crystal violet. The migratory cells on the lower side of the chamber were quantified using an inverted microscope at 100x magnification (Nikon, Japan).

### 2.8 Statistical analysis

All experiments were repeated at least for three times. SPSS 20.0 software (IBM Corp.) was used for statistical analyses. All results are expressed as the mean *±* standard deviation (SD) from three independent experiments. We used D method of normality test (Kolmogorov-Smirnov test) to test the normality of the data in SPSS. Unpaired Student’s t-test was used to compare differences between two groups. *Statist*ically significant differences among multiple groups were determined using one-way analysis of variance (ANOV*A)* followed by Tukey’s post hoc test. P < 0.05 was defined as statistically significant.

## 3. Results

### 3.1 Hypoxia promotes HRVEC viability and migration.

**Figure 1.**
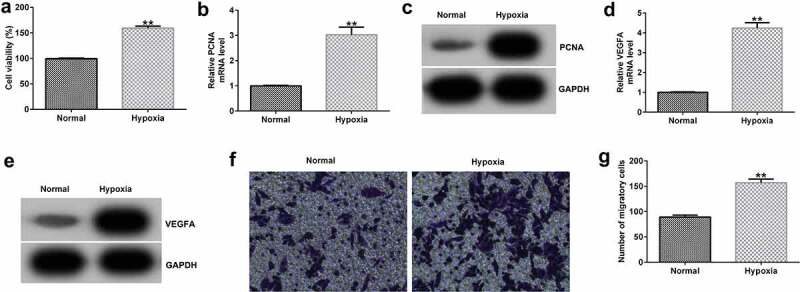
Effects of hypoxia on HRVEC viability and migration. HRVECs were exposed to hypoxia for 24 h and then cell viability and migration were determined using MTT and transwell assays. (a) Cell viability was measured using the MTT assay. (b) RT-qPCR analysis of PCNA expression. (c) PCNA expression was evaluated by western blot. (d) RT-qPCR analysis of VEGFA expression. (e) VEGFA expression was evaluated by western blot. (f) cell migration of HRVECs in different groups. (g) number of migrated cells. **P < 0.01 vs. Normal

To confirm whether the hypoxic condition worked, we firstly investigated the effects of hypoxia on HRVEC viability and migration, and VEGFA expression. HRVECs were cultured in hypoxia (1% O_2_, 5% CO_2_, 94% N_2_) for 24 h, and then cell viability and migration were determined using MTT and Transwell assays. Results indicated that compared with the normal group, hypoxia significantly enhanced HRVEC viability (Figure 1(a)), promoted PCNA (Figure 1(b,c)) and VEGFA (Figure 1(d,e)) mRNA and protein expressions, and increased HRVEC migration (Figure 1(f,g)). The data indicated that the hypoxic condition we prepared really worked.

### 3.2 Effects of CYP2J2 on Dll4 and Jagged1 expression in hypoxia-induced retinopathy.

**Figure 2.**
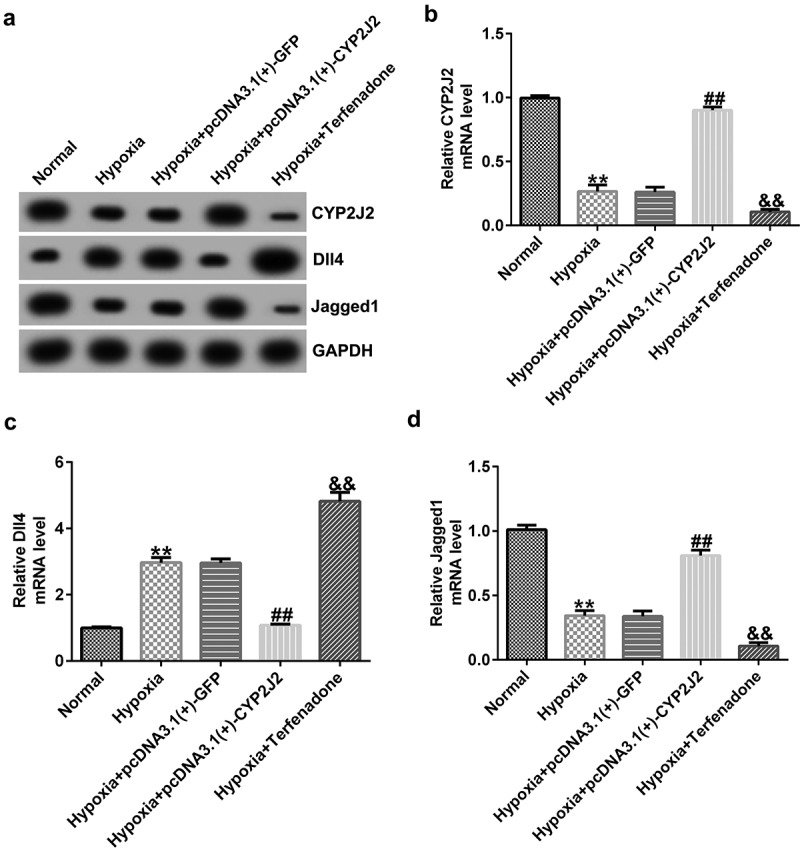
Effects of CYP2J2 or TERFENADONE on Dll4 and Jagged1 expression in a hypoxia model. HRVECs were exposed to hypoxia for 24 h and then transfected with pcDNA3.1(+)-GFP or pcDNA3.1(+)-CYP2J2, or induced by Terfenadone for 24 h. (a) western blot analysis of CYP2J2, Dll4, and Jagged1 expression. (b-d) mRNA levels of CYP2J2, Dll4, and jagged1 in different groups. **P < 0.01 vs. Normal; ##P < 0.01 vs. Hypoxia+pcDNA3.1(+)-GFP; && P < 0.01 vs. Hypoxia

To investigate the influence of CYP2J2 on Notch signaling in a hypoxia-induced ROP model, HRVECs were exposed to hypoxia for 24 h and transfected with pcDNA3.1(+)-GFP or pcDNA3.1(+)-CYP2J2. Induced cells were treated with a CYP2J2 inhibitor (Terfenadone) for 24 h. Subsequently, RT-qPCR and Western blot analysis was performed to evaluate CYP2J2, Dll4, and Jagged1 expression in different groups. We found that pcDNA3.1(+)-CYP2J2 promoted CYP2J2 and Jagged1 expression while Dll4 was down-regulated in CYP2J2-stimulated HRVECs when compared to cells treated with hypoxia+pcDNA3.1(+)-GFP. Moreover, an opposite effect was observed in cells treated with hypoxia+Terfenadone, as confirmed by reduced CYP2J2 expression, increased Dll4 expression, and reduced Jagged1 expression (Figures 2a and 1d), suggesting that CYP2J2 promotes Jagged1 expression, while down-regulating Dll4 expression in a HRVEC hypoxia-induced retinopathy model.

### 3.3 Effects of CYP2J2 on HRVEC viability and migration in hypoxia-induced retinopathy.

**Figure 3.**
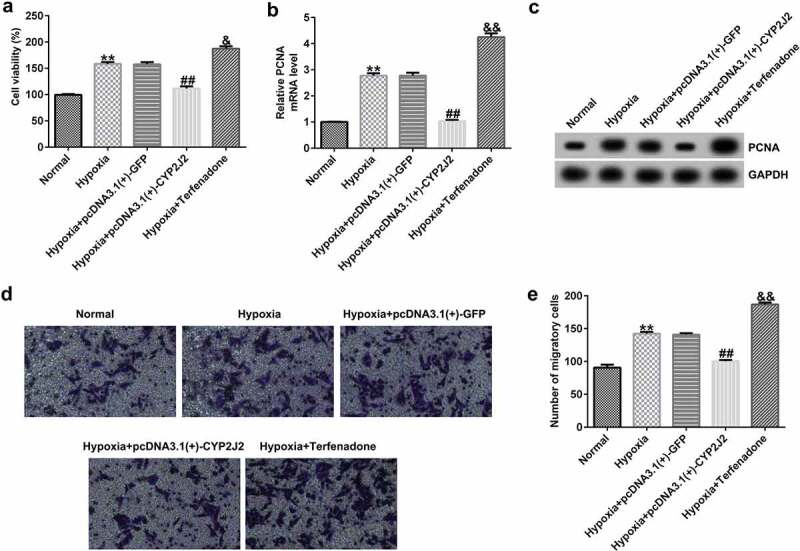
Effects of CYP2J2 or Terfenadone on HRVEC viability and migration in hypoxia-induced retinopathy. HRVECs were exposed to hypoxia for 24 h and then transfected with pcDNA3.1(+)-GFP or pcDNA3.1(+)-CYP2J2, or induced by terfenadone for the indicated time. (a) cell viability was measured using the MTT assay. (b) RT-qPCR analysis of PCNA expression. (c) PCNA expression was evaluated by western blot. (d) cell migration of HRVECs in different groups. (e) number of migrated cells. **P < 0.01 vs. Normal; ##P < 0.01 vs. Hypoxia+pcDNA3.1(+)-GFP; && P < 0.01 vs. Hypoxia

To further investigate the influence of CYP2J2 on HRVEC proliferation and migration in a hypoxia-induced model of ROP, MTT and Transwell assays were performed to evaluate cell viability and migration in different groups. As shown in (Figure 3(a-c)), hypoxia promoted HRVECs viability and enhanced PCNA expression at the protein and mRNA levels. Meanwhile, as compared to the hypoxia+pcDNA3.1(+)-GFP group, hypoxia+pcDNA3.1(+)-CYP2J2 inhibited viability and reduced PCNA expression; however, opposite results were observed following Terfenadone treatment. In addition, similar findings were observed by Transwell assay, as confirmed by the inhibited migration of HRVECs in the hypoxia+pcDNA3.1(+)-CYP2J2 group, which was promoted by hypoxia. Moreover, we found that HRVEC migration was induced by Terfenadone when compared to the hypoxia group (Figure 3(d-e)), indicating that CYP2J2 inhibits HRVEC viability and migration in under hypoxia.

### 3.4 CYP2J2 inhibits Notch signaling in an in vitro hypoxia-induced retinopathy model.

**Figure 4.**
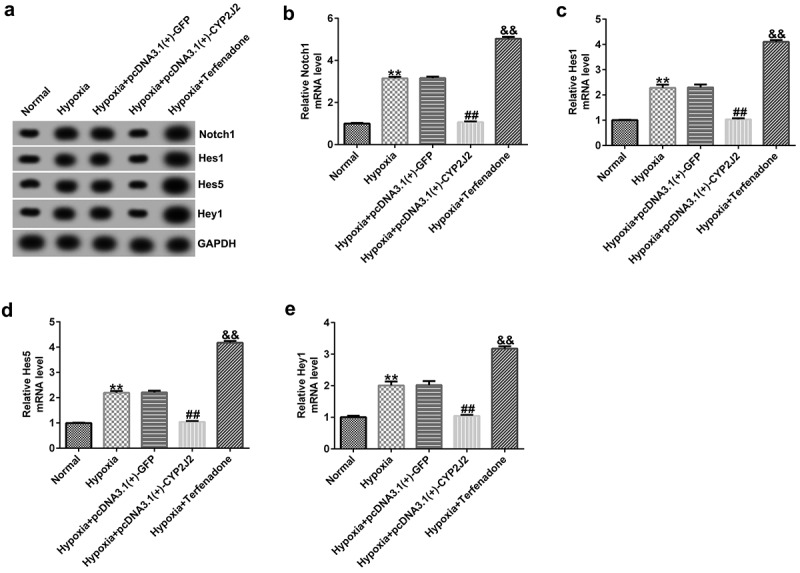
Effects of CYP2J2 on Notch signaling in hypoxia-induced retinopathy. HRVECs were exposed to hypoxia for 24 h and then transfected with pcDNA3.1(+)-GFP or pcDNA3.1(+)-CYP2J2, or treated with terfenadone. (a) western blot analysis of notch1, Hes1, Hes5, and Hey1 expression. Notch1 (b), Hes1 (c), Hes5 (d), and Hey1 (e) mRNA levels in different groups as determined by RT-qPCR. **P < 0.01 vs. Normal; ##P < 0.01 vs. Hypoxia+pcDNA3.1(+)-GFP; && P < 0.01 vs. Hypoxia

Next, we explored the regulatory roles of CYP2J2 in the Notch signaling pathway in our hypoxia-induced retinopathy model. HRVECs were exposed to hypoxia for 24 h and then transfected with pcDNA3.1(+)-GFP or pcDNA3.1(+)-CYP2J2, or treated with Terfenadone. Subsequently, RT-qPCR and Western blotting was used to determine the expression levels of genes involved in the Notch signaling pathway in different groups. These results demonstrate that hypoxia notably enhanced the expression of genes in the Notch signaling pathway, including Notch1, Hes1, Hes5, and Hey1 when compared to the control group (Figure 4(a-e)). Moreover, Notch1, Hes1, Hes5, and Hey1 were down-regulated in the hypoxia+pcDNA3.1(+)-CYP2J2 group when compared to the hypoxia+pcDNA3.1(+)-GFP group. However, opposite results were observed following Terfenadone treatment. Thus, these results reveal that CYP2J2 inhibits Notch signaling in an *in vitro* hypoxia-induced retinopathy model.

### 3.5 N-[N-(3,5-difuor-ophenacetyll-alanyl)]-S-phenylglycine t-butylester (DAPT) promotes the effects of CYP2J2 on Dll4 and Jagged1 expression in hypoxia-induced HRVECs.

**Figure 5.**
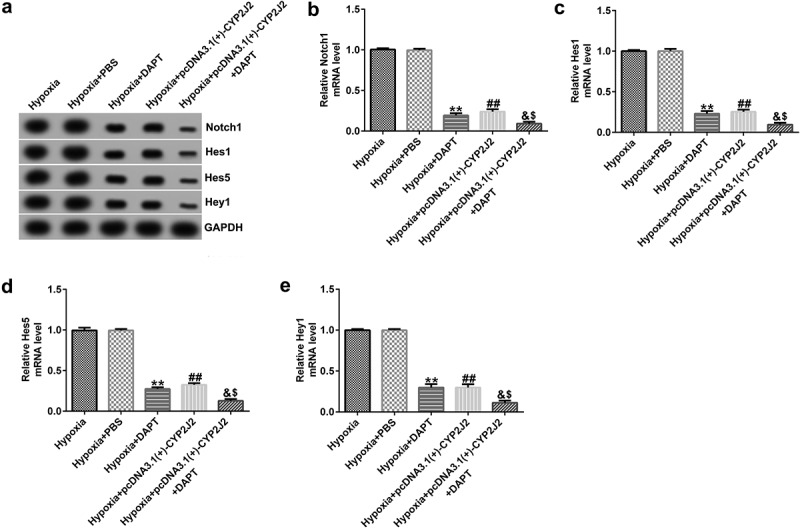
Effects of DAPT and CYP2J2 on Notch signaling in hypoxia-stimulated retinopathy. HRVECs were exposed to hypoxia for 24 h and then transfected with pcDNA3.1(+)-CYP2J2, and/or treated with DAPT. (a) Western blot analysis of Notch1, Hes1, Hes5, and Hey1 expression. Notch1 (b), Hes1 (c), Hes5 (d), and Hey1 (e) mRNA levels in different groups as determined by RT-qPCR. **P < 0.01 vs. Hypoxia+PBS; ##P < 0.01 vs. Hypoxia; &P < 0.05 vs. Hypoxia+DAPT; $P < 0.05 vs. Hypoxia+pcDNA3.1(+)-CYP2J2

To better understand the role of Notch signaling in hypoxia-induced retinopathy, HRVECs cells were exposed to hypoxia for 24 h, transfected with pcDNA3.1(+)-CYP2J2, and treated with DAPT. Subsequently, RT-qPCR and Western blotting was used to assess the expression levels of genes in the Notch signaling pathway in different groups. We observed that DAPT reduced the expression of Notch1, Hes1, Hes5, and Hey1 at both the protein (Figure 5(a)) and mRNA levels (Figure 5(b-e)). Additionally, compared to the hypoxia group, Notch1, Hes1, Hes5, and Hey1 were down-regulated in the hypoxia+pcDNA3.1(+)-CYP2J2 group, and were further down-regulated in the hypoxia+pcDNA3.1(+)-CYP2J2+ DAPT group. Our findings suggest that DAPT promots the effects of CYP2J2 on Notch signaling in hypoxia-induced HRVECs.

### 3.6 DAPT promotes the effects of CYP2J2 on cell viability and migration in hypoxia-induced HRVECs.

**Figure 6.**
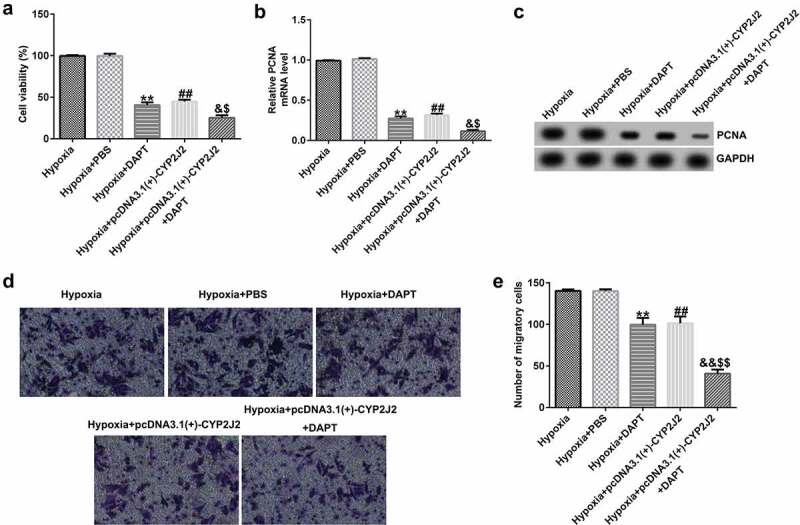
Effects of DAPT and CYP2J2 on cell viability and migration in hypoxia-induced retinopathy. HRVECs were exposed to hypoxia for 24 h and then transfected with pcDNA3.1(+)-CYP2J2, and/or treated with DAPT. (a) MTT assay was used to evaluate cells viability. (b) PCNA expression as determined by western blot. (c) RT-qPCR analysis of PCNA expression. (d) Migration of HRVECs in different groups. (e) Number of migrated cells. **P < 0.01 vs. Hypoxia+PBS; ##P < 0.01 vs. Hypoxia; &, &&P < 0.05, 0.01 vs. Hypoxia+DAPT; $^,^ $$P < 0.05, 0.01 vs. Hypoxia+pcDNA3.1(+)-CYP2J2

To further elucidate the role of DAPT in hypoxia-induced retinopathy, HRVECs were exposed to hypoxia for 24 h, transfected with pcDNA3.1(+)-CYP2J2, and treated with DAPT. Results revealed that DAPT markedly inhibited the viability of HRVECs (Figure 6(a)) and reduced PCNA expression at the protein and mRNA levels (Figure 6(b,c)) when compared to the hypoxia+PBS group. Furthermore, we found that pcDNA3.1(+)-CYP2J2 inhibited HRVEC viability and PCNA expression at both the protein and mRNA levels. These results were further exacerbated in the hypoxia+pcDNA3.1(+)-CYP2J2+ DAPT group. Meanwhile, as shown in (Figure 6(d,e)), HRVECs migration was inhibited in the hypoxia+DAPT and hypoxia+pcDNA3.1(+)-CYP2J2 group. Additionally, this reduction was exacerbated by DAPT, revealing that DAPT promotes the inhibitory effect of CYP2J2 on cell viability and migration in hypoxia-induced HRVECs. Thus, our findings suggest that DAPT promotes the effects of CYP2J2 on cell viability and migration in hypoxia-induced HRVECs.

### 3.7 EDTA reverses the inhibitory effect of CYP2J2 on Notch signaling in hypoxia-induced HRVECs.

**Figure 7.**
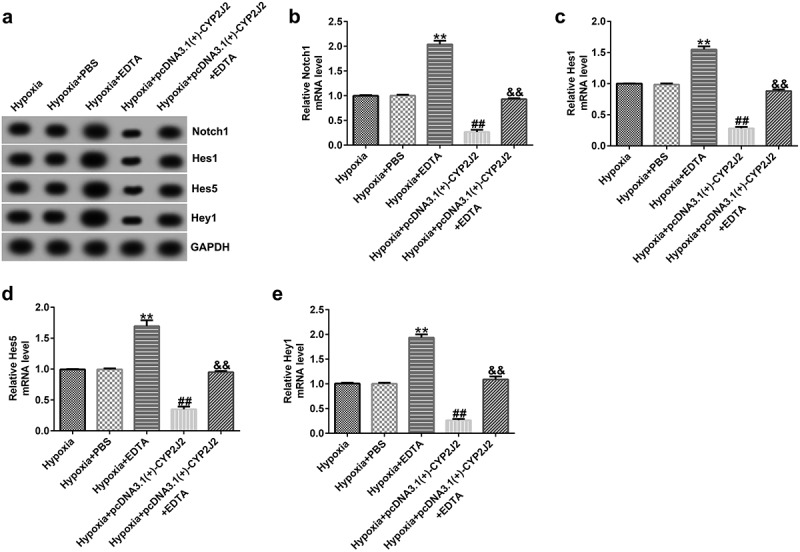
Effects of EDTA and CYP2J2 on Notch signaling in hypoxia-induced retinopathy. HRVECs were exposed to hypoxia for 24 h and then transfected with pcDNA3.1(+)-CYP2J2, and/or treated with EDTA. (a) Western blot analysis of Notch1, Hes1, Hes5, and Hey1 expression. Notch1 (b), Hes1 (c), Hes5 (d), and Hey1 (e) mRNA levels in different groups as determined by RT-qPCR . **P < 0.01 vs. Hypoxia+PBS; ##P < 0.01 vs. Hypoxia; &&P < 0.05 vs. Hypoxia+pcDNA3.1(+)-CYP2J2

Having explored the relationship between Notch1 inhibition and CYP2J2, we next explored the mechanisms of a Notch1 agonist and CYP2J2 in hypoxia-induced retinopathy. HRVECs cells were exposed to hypoxia for 24 h, transfected with pcDNA3.1(+)-CYP2J2, and treated with EDTA. As shown in (Figure 7(a)), EDTA notably enhanced the expression of genes related to the Notch signaling pathway, including Notch1, Hes1, Hes5, and Hey1. Meanwhile, we observed an opposite effect in the pcDNA3.1(+)-CYP2J2 group when compared to hypoxia. However, this reduction was reversed by the addition of EDTA. Additionally, we found similar results on the mRNA levels of Notch1, Hes1, Hes5, and Hey1, as shown in (Figure 7(b-e)). Together, these findings suggest that EDTA reverses the inhibitory effect of CYP2J2 on the Notch signaling pathway in hypoxia-induced retinopathy.

### 3.8 EDTA reverses the inhibitory effect of CYP2J2 on cell viability and migration in hypoxia-induced retinopathy.

**Figure 8.**
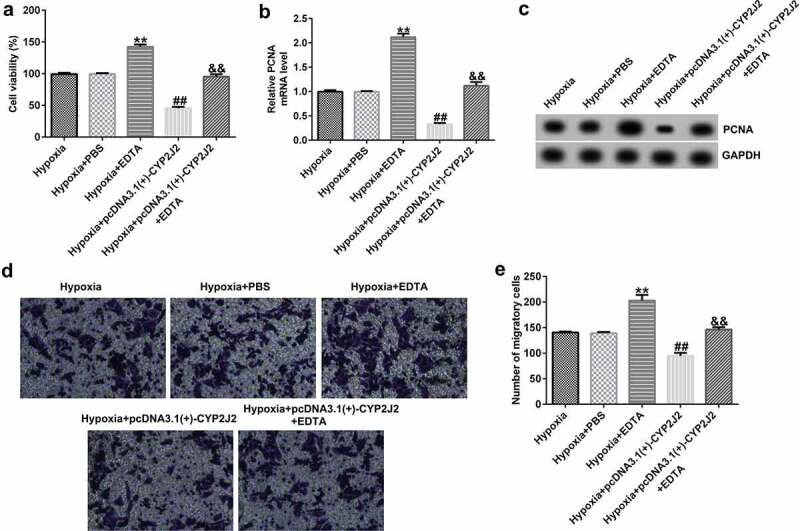
Effects of EDTA and CYP2J2 on cell viability and migration in hypoxia-induced retinopathy. HRVECs were exposed to hypoxia for 24 h and then transfected with pcDNA3.1(+)-CYP2J2, and/or treated with EDTA. (a) Cell viability was measured by MTT assay. (b) PCNA expression as determined by western blot. (c) RT-qPCR analysis of PCNA expression. (d) Migration of HRVECs in different groups. (e) number of migrated cells. **P < 0.01 vs. Hypoxia+PBS; ##P < 0.01 vs. Hypoxia; &&P < 0.05 vs. Hypoxia+pcDNA3.1(+)-CYP2J2

Finally, we evaluated the effects of EDTA and CYP2J2 on cell viability and migration in hypoxia-induced retinopathy. HRVECs were exposed to hypoxia for 24 h, and then transfected with pcDNA3.1(+)-CYP2J2 or treated with EDTA. As presented in (Figure 8(a)), EDTA markedly increased the viability of HRVECs and enhanced PCNA expression at the protein and mRNA levels (Figure 8(b-c)) when compared to the hypoxia+PBS group. In addition, after pcDNA3.1(+)-CYP2J2 transfection, HRVEC proliferation was reduced and PCNA expression was reduced at the protein and mRNA levels. However, these effects were reversed by the addition of EDTA. Further, a Transwell assay revealed that pcDNA3.1(+)-CYP2J2 inhibited HRVEC migration, which could be rescued by the addition of EDTA (Figure 8(d-e)). These data reveal that EDTA reverses the inhibitory effect of CYP2J2 on cell viability and migration in hypoxia-induced retinopathy.

## 4. Discussion

The novel findings of our research are as follows: i) CYP2J2 inhibits HRVEC viability and angiogenesis in a hypoxia-induced retinopathy model; ii) the effects of CYP2J2 on hypoxia-induced HRVEC are regulated via the Notch signaling pathway. To our knowledge, this work represents the first analysis and mechanistic understanding of the role of CYP2J2 in hypoxia-induced HRVECs. The schematic diagram of summary of this study was presented in Supplementary Figure 1.

Retinopathy of prematurity, a type of retinal neovascularization in preterm infants, has causes retinal vascular occlusion or growth arrest under hyperoxia, and neovascularization after return to normoxia [20]. Additionally, severe cases may result in retinal proliferation, detachment, and even blindness. The survival rate of premature babies with ROP is improving due improved medical treatment, including the use of anti-VEGF drugs and laser-based therapies. Wang et al. reported the use of anti-VEGF drugs for treatment of ROP [21]. Additionally, Ekinci et al. have suggested comparing the efficacy between intravitreal aflibercept and laser photocoagulation in ROP treatment [22]. However, anti-VEGF drugs may also produce adverse reactions such as endophthalmitis, fail to completely prevent retinal neovascularization, and aggravate neurodegeneration [7]. Therefore, there is an urgent need to explore safer and more effective agents to inhibit angiogenesis.

Lipids are involved in the regulation of neovascular choroid disease. CYP2J2, a member of the CYP450 oxidase family, is highly expressed in the cardiovascular system and is involved in regulating blood vessels. Wang et al. reported that CYP2J2 gene polymorphism can affect the occurrence and development of ischemic stroke by modulating transcriptional activity [9]. In addition, Zhao et al. found that endothelium-specific CYP2J2 overexpression improves cardiac dysfunction by promoting angiogenesis via Jagged1/Notch1 signaling [23]. We speculated that CYP2J2 may also be highly expressed in HRVECs and regulate retinal neovascularization. In this study, HRVECs were exposed to hypoxia for 24 h to establish an *in vitro* retinopathy model. Previous reports have suggested that Dll4 and Jagged1 play an important role in angiogenesis. Marchetto et al. revealed that endothelial Jagged1 antagonizes Dll4/Notch signaling in decidual angiogenesis during early mouse pregnancy [24]. Oon suggested the role of Dll4 in Jagged1-induced tumor angiogenesis and tumor growth [25]. Based on these findings, RT-qPCR and Western blot analysis were performed to evaluate CYP2J2, Dll4, and Jagged1 levels in different groups. We found that up-regulation of CYP2J2 notably promoted CYP2J2 and Jagged1 expression, while Dll4 was down-regulated in CYP2J2-stimulated HRVECs when compared to the hypoxia+pcDNA3.1(+)-GFP group; however, opposite results were observed following Terfenadone treatment in hypoxia-induced HRVECs.

To further investigate the effects of CYP2J2 on HRVEC viability and migration, we evaluated HRVEC viability and migration in different groups using MTT and Transwell assays. Our data revealed that up-regulation of CYP2J2 inhibited HRVEC viability, reduced the expression of PCNA, and inhibited migration; however, Terfenadone induced opposite effects on HRVEC viability and migration in hypoxia-induced retinopathy. Together, these findings confirmed that CYP2J2 inhibits viability and angiogenesis of HRVECs in an *in vitro* model of hypoxia-induced retinopathy. However, as overexpression of CYP2J2 significantly inhibited cell viability, whether it is possible that the decreased number of migratory cells using transwell assays is a result of the down-regulated cell viability remains to be explored. Besides, in this study, we only used Terfenadone to study the effects of CYP2J2 down-regulation on hypoxia-induced HRVECs. Loss-of-function experiments using shRNAs or siRNAs specifically targeting CYP2J2 were not performed. This was a limitation of current study.

In the central nervous system, Notch signaling plays an important role in regulating angiogenesis. Fan et al. found that the level of Dll4 in a middle cerebral artery occlusion model was positively correlated with brain cell apoptosis in the cerebral infarct area of rats while also being significantly negatively correlated with the number of new blood vessels [26]. Zhai et al. found that angiogenesis was increased and the Notch1 signal was activated when brain VECs were co-cultured with astrocytes [27]. We determined the levels of Notch signaling pathway related genes in different groups using RT-qPCR and Western blotting. We observed that Notch1, Hes1, Hes5, and Hey1 were down-regulated in the hypoxia+pcDNA3.1(+)-CYP2J2 group when compared to the hypoxia+pcDNA3.1(+)-GFP group. However, these data were opposite in the Terfenadone treatment group, suggesting that CYP2J2 inhibits Notch signaling in hypoxia-induced retinopathy.

To further investigate whether the mechanism by which CYP2J2 inhibits the growth of retinal neovascularization is related to the Notch signaling pathway, HRVECs were exposed to hypoxia for 24 h and then transfected with pcDNA3.1(+)-CYP2J2 or treated with DAPT or EDTA. Results from RT-qPCR and Western blotting revealed that DAPT promoted the effects of CYP2J2 on Notch signaling in hypoxia-induced retinopathy while EDTA reversed the inhibitory effect of CYP2J2 on Notch signaling in hypoxia-induced retinopathy. Further analysis using MTT and Transwell assays suggested that DAPT promotes the effects of CYP2J2 on cell viability and migration in hypoxia-induced retinopathy while EDTA reverses the inhibitory effect of CYP2J2 on cell viability and migration in hypoxia-induced retinopathy. However, this study did not conduct *in vivo* experiments, which is another limitation of our study. We will explore this in depth in future research.

## 5. Conclusions

CYP2J2 inhibits the viability and angiogenesis of retinal vascular endothelial cells via regulating the Notch signaling pathway in a hypoxia-induced retinopathy model. Therefore, it is expected to be a promising avenue for retinopathy therapy.

## Supplementary Material

Supplemental Material

## Data Availability

The datasets used and/or analyzed during the current study are available from the corresponding author upon reasonable request.

## Highlights

CYP2J2 inhibits HRVEC viability and migration in hypoxia-induced retinopathy.

CYP2J2 inhibits Notch signaling in an *in vitro* hypoxia-induced retinopathy model.

EDTA reverses the inhibitory effect of CYP2J2 on cell viability and migration in hypoxia-induced retinopathy.
