# Supplementary material for: Cytochrome P450 2J2 inhibits the proliferation and angiogenesis of retinal vascular endothelial cells by regulating the Notch signaling pathway in a hypoxia-induced retinopathy model
Source: Bioengineered. 2021 Nov 30;12(2):10878–90. doi: 10.1080/21655979.2021.1994722 (PMC8809993; doi:10.1080/21655979.2021.1994722)
Supplement: Supplemental Material [file KBIE_A_1994722_SM3706.zip › supplementary figure caption.docx]

**Supplementary Figure 1**

The schematic diagram of summary of this study.
